# Effect of medium with moderate temperature on patient's body temperature during percutaneous endoscopic lumbar discectomy

**DOI:** 10.1186/s13018-022-03226-x

**Published:** 2022-06-28

**Authors:** Maji Sun, Fuchao Chu, Lidong Zhang, Rui Zhao, Xiaona Liu, Huilin Yu, Bin Pan, Jibin Wu, Feng Yuan

**Affiliations:** Department of Spine Surgery, The Affiliated Hospital of Xuzhou Medical University, Xuzhou, 221006 Jiangsu People’s Republic of China

**Keywords:** PELD, Hypothermia, Inflammatory cytokines, Inflammatory reaction

## Abstract

**Purpose:**

To explore the influence of irrigating fluid at different temperatures on patients' body temperature and local inflammatory mediators during spinal endoscopy.

**Methods:**

110 cases of intervertebral foramen surgery in our hospital from January 2019 to October 2021 were randomly divided into control group and observation group. Operations of both groups were performed by the same experienced chief physician. The observation group was irrigated with 37 °C constant temperature saline, while the control group was irrigated at room temperature. The effect was evaluated by monitoring the intraoperative temperature, postoperative VAS score and the levels of inflammatory factors, such as TNF-α, IL-1, IL-6 and IL-10 in drainage fluid.

**Results:**

After 30 min of operation, overall temperature of the control group dropped significantly, and 50 cases (90.9%) had hypothermia, *P* < 0.05. There was no significant difference in preoperative VAS score between the two groups *P* > 0.05. The VAS score of observation group was significantly lower than that of control group at 6 h and 1 month after operation, *P* < 0.05. At 0, 3 and 6 h after operation, the values of TNF-α, IL-1, IL-6 and IL-10 in the observation group were significantly lower than those in the control group (*P* < 0.05).

**Conclusions:**

Isothermal flushing solution can reduce the incidence of hypothermia and effectively alleviate local inflammatory reaction.

## Introduction

Lumbar disc herniation (LDH) is becoming more and more common as degenerative disease of lumbar spine in clinic [1–3]. The symptoms of nerve root compression such as lumbago and radiation pain of lower limbs caused by LDH can seriously affect the quality of life of patients [4]. LDH can be treated by conservative, interventional or surgical treatment, among which surgical treatment is commonly used, including traditional open surgical methods, such as simple discectomy, microendoscopic discectomy (MED) and spinal fusion. Due to the precise operation, MED provides a better operation area and less damage to surrounding tissues. In recent years, minimally invasive surgery, represented by transforaminal endoscopic spine system (TESSYS), has attracted extensive attention in the treatment of lumbar disc herniation, especially in the treatment of lumbar disc herniation with nerve root canal stenosis, which has been widely recognized clinically [4–8]. Percutaneous endoscopic lumbar discectomy (PELD) operation is superior to traditional operation in tissue damage, bleeding volume and recovery period. However, in PELD operation, a large amount of room temperature flushing fluid is needed to expose the visual field, which often leads to hypothermia in patients during operation. Although mild hypothermia can reduce the metabolic rate and oxygen consumption of the body, it has a protective effect on the body. However, prolonged hypothermia will increase the oxygen consumption of the body, lead to increased CO2, acidosis and accelerated metabolism, which will affect the postoperative therapeutic effect [9, 10]. The purpose of this study is to explore whether hypothermia events can be prevented by heating the normal saline flushing solution with a thermostat to 37 °C during the treatment of lumbar disc herniation with PELD.

## Materials and methods

### Study design

A prospective, randomized, single blind, single-center clinical study was adopted. 110 patients who underwent intervertebral foramen surgery from January 2019 to October 2021 were selected and randomly divided into the control group and the observation group according to the digital random method. Both groups were operated by experienced doctors with senior professional titles. All steps in the experiment comply with Helsinki Declaration and are approved by the Ethics Committee of Affiliated Hospital of Xuzhou Medical University.

### General information

There were 33 males and 22 females in the control group, aged 33–58 years with an average age of 41.5 ± 0.9 years, average height is 172.31 ± 2.17 cm and the average weight is 65.34 ± 7.69 kg. In the experiment group, there were 41 males and 14 females, aged 31–57 years, with an average age of 40.8 ± 0.8 years, an average height of 173.12 ± 1.47 cm and an average weight of 64.79 ± 8.77 kg. There was no significant difference in baseline data between the two groups (*P* > 0.05), which was comparable. Normal saline at room temperature (25–27 °C) was used as irrigation liquid in the control group. The normal saline used in the observation group was heated to 37 °C in the operating room thermotank before operation. During the operation, the thermostatic infusion set was installed on the infusion tube, and heating temperature was set at 37 °C. Thermostatic infusion set was checked regularly to ensure its effective work.

Inclusion criteria: (1) Lumbar disc herniation was diagnosed by CT and MRI without calcification or slight calcification; (2) Conservative treatment is ineffective; (3) Complete clinical data.

Exclusion criteria: (1) LDH combined with lumbar instability, bilateral nerve root symptoms, severe lumbar deformity, lumbar spinal stenosis and spinal tumor; (2) Complicated with major organ dysfunction, coagulation dysfunction, severe osteoporosis and metabolic osteopathy; (3) Pregnant or lactating women; (4) Patients with mental illness.

### Surgical method

Preoperative preparation: The operating room temperature is 23 °C, and intravenous fluids are infused at normal temperature. 3000 ml normal saline was used as intraoperative flushing fluid. The control group used normal saline at room temperature, while the observation group used normal saline at 37 °C.

Patients with lumbar disc herniation were treated with transforaminal endoscopic spine system (TESSYS) technique. Before operation, patients were placed in lateral position, the affected side was located above, and lower part is padded with a posture pad to enlarge the intervertebral foramen of the affected side. Mark the posterior midline and crista iliaca under the fluoroscopy of C-arm X-ray machine. 1% lidocaine was used for local infiltration anesthesia at the puncture site, 18-gauge puncture needle was used to enter the puncture site, and 2 ml of 0.5% lidocaine was injected for local anesthesia of the tissues around the articular process. Inserting a guide wire, making an incision of about 8 mm around the puncture point, inserting a guide rod and a sleeve along the guide wire, gradually expanding the intervertebral foramen, inserting a working sleeve, placing the sleeve under the nerve root, inserting a nucleus pulposus clamp and a basket clamp, taking out the prominent intervertebral disc and proliferative ligamentum flavum of the lateral recess, and loosening the compressed nerve root. Patients with hyperosteogeny are treated with abrasive drill under microscope. Using dual-frequency radio frequency electrode, the tear of the annulus fibrosus was crimped and formed. Then, electrocoagulation was used to stop bleeding, seam-free adhesive tape was used to bond the incision, and dressing was used to cover the incision.

### Data collection

Temperature monitoring: During the operation, medical staff used mercury thermometer to measure and record the core body temperature of patients within 60 min and every 15 min. Choose anal temperature to measure, just apply lubricant to one end of mercury thermometer, insert it into anus for about 1/2, and take out the reading after 5 min.

VAS Grade: Visual Analogue Scale (VAS) scoring method mainly quantifies patients' subjective pain feeling, which is a scoring method for evaluating the degree of pain. The visual analog ruler uses a straight line without division, and one end of the horizontal line is 0, which means no pain; The other end is 10, indicating severe pain. VAS scores were given to patients before, during and 1 month after operation.

Inflammatory cytokines detection: Use sterile syringes collected postoperative 0 h, 3 h and 6 h of drainage fluid. The samples are stored in the frozen storage box and quickly taken to the laboratory. First, centrifuge at 4 °C with 1500*g* for 15 min, and then centrifuge at 3000*g* for 15 min after taking the supernatant. After twice centrifugation, the supernatant was stored in a refrigerator at − 80 °C. The levels of TNF-α, IL-1, IL-6 and IL-10 in drainage fluid were detected step by step according to the instructions of ELISA reagent.

### Statistical method

SPSS23.0 statistical software is used for statistical processing, and those who meet the normal distribution in the measurement data are represented by ($\overline{x} \pm s$). The data are first tested for normality and homogeneity of variance, and independent sample T test is used for comparison between groups. Chi-square test was used to compare the counting data of hypothermia, chills and adverse cardiovascular events. *P* < 0.05 is statistically significant.

## Result

### Temperature monitoring results

**Fig. 1:**
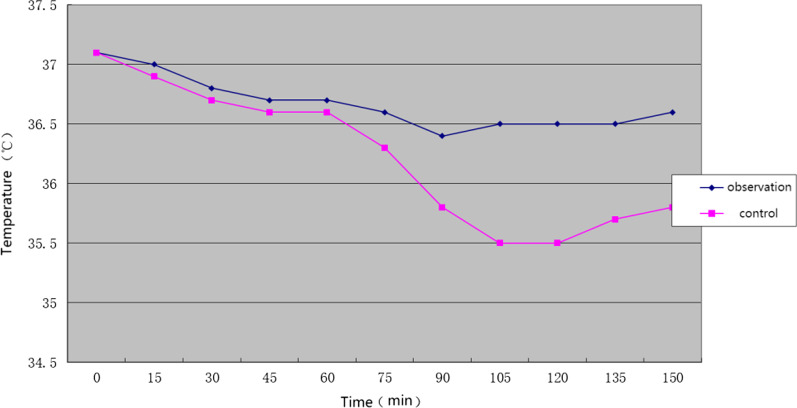
Comparison of body temperature of patients in observation group and control group in different periods of operation ($\overline{X} \pm S$, *N* = 55)

The temperature of observation group and control group were monitored every 15 min after anesthesia induction. Within 60 min after anesthesia induction, there was no significant difference in anal temperature between observation group and control group recorded at each monitoring timepoint (*P* > 0.05). After 60 min, the temperature of the control group dropped significantly with time, the lowest temperature was 35.5 ± 0.3 °C, and the lowest temperature of the observation group was 36.3 ± 0.4 °C, the difference between the two groups was statistically significant (*P* < 0.05). Among the 55 cases in the observation group, 20 cases with hypothermia (20/55, 36.4%) were significantly lower than those in the control group (50/55, 90.9%), and the difference was statistically significant (*P* < 0.05) (Fig. 1).

### Comparison of VAS scores between two groups before and after operation

The preoperative pain degree (VAS) of patients was 7.41 ± 4.01 in the observation group and 7.36 ± 3.77 in the control group, and there was no significant difference (*P* > 0.05). Six hours after operation, the VAS score of the observation group was 3.79 ± 2.13, which was significantly lower than that of the control group 4.18 ± 2.35, and the difference was statistically significant (*P* < 0.05). One month after operation, the VAS score of the observation group was 1.74 ± 0.61 significantly lower than that of the control group 2.21 ± 0.79, and the difference was statistically significant (*P* < 0.05) (Table 1).

**Table 1:** Comparison of VAS scores between two groups before and after operation (X ± S, *N* = 55)

Group	Case number	VAS grade
Control group		
Preoperative	55	7.36 ± 3.77
6 h after operation	55	4.18 ± 2.35*
1 month after operation	55	2.21 ± 0.79*
Observation group		
Preoperative	55	7.41 ± 4.01
6 h after operation	55	3.79 ± 2.13*^#^
1 month after operation	55	1.74 ± 0.61*

Compared with this group before operation (*) *p* < 0.05; compared with the control group (#) *p* < 0.05

### Levels of inflammatory cytokines TNF-α, IL-1, IL-6 and IL-10 in drainage fluid of two groups

At 0 h after operation, the values of TNF-α in the observation group was 71.5 ± 17.8 significantly lower than that of the control group 87.6 ± 21.3, and the values of IL-1 in the observation group was 121.5 ± 24.8 significantly lower than that of the control group 147.1 ± 23.5. The values of IL-6 in the observation group was 6711.5 ± 985.4 significantly lower than that in the control group 7567.4 ± 1027.3, and the values of IL-10 in the observation group was 197.3 ± 54.3 significantly lower than that in the control group 220.4 ± 43.8. Three hours later, the values of TNF-α in observation group was 90.4 ± 18.6 significantly lower than that in control group 107.5 ± 19.3, and values of IL-1 in observation group was 154.9 ± 33.4 significantly lower than that in control group 185.9 ± 31.3. The values of IL-6 in the observation group was 8907.3 ± 1278.9 significantly lower than that in the control group 11,107.5 ± 1146.7, and the values of IL-10 in the observation group was 378.6 ± 49.7 significantly lower than that in the control group 423.1 ± 56.7. Six hours after operation, the values of TNF-α in the observation group was 98.7 ± 19.3 significantly lower than that of the control group 131.4 ± 21.8, and the values of IL-1 in the observation group was 169.1 ± 28.7 significantly lower than that of the control group 207.6 ± 33.4. The values of IL-6 in the observation group was 10,786.9 ± 1344.3 significantly lower than that in the control group 13,211.5 ± 1223.1, and the values of IL-10 in the observation group was 460.7 ± 67.4 significantly lower than that in the control group 517.5 ± 66.1 (Table 2).

**Table 2:** Values of inflammatory factors in drainage fluid (pg/ml)

	Case number	0 h after operation	3 h after operation	6 h after operation
TNF-α control	55	87.6 ± 21.3	107.5 ± 19.3	131.4 ± 21.8
Observation	55	71.5 ± 17.8*	90.4 ± 18.6*	98.7 ± 19.3*
*t*		3.712	2.117	3.762
*p*		0.031	0.027	0.017
IL-1 control	55	147.1 ± 23.5	185.9 ± 31.3	207.6 ± 33.4
Observation	55	121.5 ± 24.8*	154.9 ± 33.4*	169.1 ± 28.7*
*t*		4.177	5.633	2.176
*p*		0.023	0.039	0.015
IL-6 control	55	7567.4 ± 1027.3	11,107.5 ± 1146.7	13,211.5 ± 1223.1
Observation	55	6711.5 ± 985.4*	8907.3 ± 1278.9*	10,786.9 ± 1344.3*
*t*		3.119	4.357	4.197
*p*		0.043	0.031	0.027
IL-10 control	55	220.4 ± 43.8	423.1 ± 56.7	517.5 ± 66.1
Observation	55	197.3 ± 54.3*	378.6 ± 49.7*	460.7 ± 67.4*
*t*		5.132	3.911	4.147
*p*		0.041	0.031	0.011

Compared with the control group (*) *p* < 0.05

## Discussion

Ensuring the clear vision during the operation is the premise for the smooth operation of intervertebral foramen, so a large amount of irrigation fluid is often used in the operation of intervertebral foramen. At present, the irrigating fluid used in intervertebral foramen endoscope surgery is mostly room temperature irrigating fluid, and the temperature is about 23 °C. However, after a large amount of irrigation fluid enters the human body, patients often suffer from chills and hypothermia during operation [11, 12]. The research shows that reasonably increasing the temperature of lavage fluid during operation can effectively avoid the occurrence of adverse events during operation, and is of great significance to the surgical treatment effect and postoperative recovery of patients. Kim [11] and others conducted a study on arthroscopic shoulder surgery, and randomly divided 50 patients who needed arthroscopic shoulder surgery into two groups: room temperature lavage group and heated lavage group. The data showed that the temperature of patients in the warming group decreased by 0.28 ± 0.2 °C on average during operation, while that of patients in the room temperature group decreased by 0.86 ± 0.2 °C on average, *P* < 0.001. Therefore, the author thinks that patients undergoing shoulder arthroscopy surgery with warming flushing solution can effectively avoid hypothermia. To study the influence of different temperature flushing solution on patients undergoing percutaneous intervertebral foramen surgery, this study monitored and compared the patient's temperature during operation, VAS score after operation and inflammatory factor level of drainage fluid. Patients in our hospital undergoing elective interforaminal endoscopic surgery were randomly divided into control group and observation group. Normal saline at room temperature (25–27 °C) was used as irrigation solution in the control group, and constant temperature saline at 37 °C was used in the observation group. During the operation, the hanging height of the liquid bag was used to control the water pressure and keep the water pressure relatively stable. During the temperature monitoring, 20 patients in the observation group (20/55, 36.4%) had hypothermia, which was significantly lower than that in the control group (50/55, 90.9%). The lowest temperature in the observation group was (36.3 ± 0.4) °C, which was significantly higher than that in the control group (35.5 ± 0.3) °C. This shows that the normal temperature irrigation solution can significantly affect the patient's body temperature, especially after 60 min of operation, the body temperature of patients in the control group has a significant downward trend, which is significantly lower than that in the observation group. The patient's body temperature of observation group remained at a relatively stable state 60 min after the operation began, and was basically stable at 36.5 °C after 100 min. This has an explanatory significance for the occurrence of hypothermia during operation, and it is confirmed that the isothermal flushing fluid can effectively reduce the incidence of hypothermia and smooth the temperature fluctuation of perioperative patients.

In the comparison of preoperative and postoperative pain scores of all patients who participated in the study, preoperative pain degree (VAS) of patients in the observation group was 7.41 ± 4.01 and that of the control group was 7.36 ± 3.77, and there was no significant difference (*P* > 0.05). Six hours after operation, the VAS score of the observation group was 3.79 ± 2.13, which was significantly lower than that of the control group 4.18 ± 2.35, and the difference was statistically significant (*P* < 0.05). One month after operation, the VAS score of the observation group was 1.74 ± 0.61 significantly lower than that of the control group 2.21 ± 0.79, and the difference was statistically significant (*P* < 0.05). It shows that the warming irrigation solution can effectively reduce the postoperative pain degree of patients undergoing intervertebral foramen endoscopic surgery, and this difference still exists even 1 month after operation. The specific reason why the water temperature can relieve the pain of patients is not clear. It may be related to the stimulation of the damaged nerves and surrounding soft tissues caused by low temperature, which needs further study.

Studies have shown that the expression of pro-inflammatory cytokines such as TNF-α, IL-1, IL-6 will be significantly increased when the body is stimulated by cold. In addition, the expression products of pro-inflammatory cytokines can induce the increase of other anti-inflammatory cytokines, such as IL-10 [13–15]. The higher the degree of inflammatory reaction, the greater the influence on the surgical trauma. At the same time, inflammatory reaction can cause dysfunction of multiple organs, and even death in severe cases [16]. As an acute stress source, normal temperature lavage fluid can cause significant changes in neuroendocrine of the body, and immune system cells release a large number of immune factors into the body to participate in the regulation of the body's immune and inflammatory response. To explore the effect of temperature stimulation on the level of local inflammatory factors, the levels of inflammatory factors in drainage fluid were recorded and compared immediately, 3 h and 6 h after operation. Taking TNF-α, IL-1, IL-6 and IL-10 as observation indexes, it was found that the observed values increased in the whole process 6 h after operation. However, at the same time, the inflammatory indexes in the observation group were significantly lower than those in the control group. This indicates that heated rinse can effectively reduce the more obvious inflammatory reaction caused by cold stimulation, and it will also play an effective role in protecting local tissues.

To sum up, the results of this experiment show that warming irrigation solution can effectively reduce the incidence of intraoperative hypothermia, reduce local inflammatory reaction and patient pain compared with room temperature irrigation solution. One limitation of this study is that it can't fully explain the mechanism and reason of VAS score reduction in the observation group after operation, which needs further study.

## Data Availability

The data sets used and/or analyzed during the current study are available from the corresponding author on reasonable request. We do not have ethical permission to upload the data set into a repository. Please note that all study data has been anonymised for confidentiality purposes.
